# A Signal Normalization Technique for Illumination-Based Synchronization of 1,000-fps Real-Time Vision Sensors in Dynamic Scenes

**DOI:** 10.3390/s100908719

**Published:** 2010-09-20

**Authors:** Lei Hou, Shingo Kagami, Koichi Hashimoto

**Affiliations:** Department of System Information Sciences, Graduate School of Information Sciences, Tohoku University, 6-6-01 Aramaki Aza Aoba, Aoba-ku, Sendai 980-8579, Japan; E-Mails: swk@ic.is.tohoku.ac.jp (S.K.); koichi@ic.is.tohoku.ac.jp (K.H.)

**Keywords:** high-speed vision, robot vision, vision chip, camera synchronization, phase-locked loop, signal processing, visible light communication, signal normalization, quadrature detection

## Abstract

To acquire images of dynamic scenes from multiple points of view simultaneously, the acquisition time of vision sensors should be synchronized. In this paper, an illumination-based synchronization derived from the phase-locked loop (PLL) mechanism based on the signal normalization method is proposed and evaluated. To eliminate the system dependency due to the amplitude fluctuation of the reference illumination, which may be caused by the moving objects or relative positional distance change between the light source and the observed objects, the fluctuant amplitude of the reference signal is normalized framely by the estimated maximum amplitude between the reference signal and its quadrature counterpart to generate a stable synchronization in highly dynamic scenes. Both simulated results and real world experimental results demonstrated successful synchronization result that 1,000-Hz frame rate vision sensors can be successfully synchronized to a LED illumination or its reflected light with satisfactory stability and only 28-*μ*s jitters.

## 1. Introduction

We have been working on the development of synchronization techniques for multiple vision sensors utilizing optical trigger information from an illumination source. Here, we refer by the term synchronization to produce temporally-aligned vision frames in image acquisition, instead of establishing correct correspondence between the vision frames. Although virtual synchronization, e.g., [1], can be employed in some applications, real synchronization is more advantageous when, for example, the motion of target objects is fast and random, and/or highly precise position information is required.

Although many of state-of-the-art vision sensors are equipped with dedicated electrical inputs/outputs for synchronization trigger signals, a major problem in this classical and widely-used means is that deployment of synchronization wires is cumbersome in some situations—short wires may impose constraints on spatial configuration of vision sensors; long wires may cause unstable synchronization. Instead of dedicated synchronization wires, some systems allow synchronization through standard electronic buses used for image transfer such as IEEE 1394 [2]. To gain more convenience, time synchronization techniques for wired or wireless communication buses and networks are sometimes exploited [3,4], with the accuracy of 1 ms and at the cost of extra network resources. Popular techniques for wireless networks [5] require implementation of time synchronization at the MAC layer for fine-grained accuracy, reporting a high precision on the order of a few microseconds. Due to this nondeterminism, it is difficult to make certain when a synchronization packet started to propagate from the sender. RBS [6] introduced a receiver-receiver synchronization scheme to remove the effect of the sender nondeterminism, producing a high precision around 1.85 *μ*s, but requires many message exchanges between receivers to achieve high precision. TPSN [7] with an accuracy of 20 *μ*s, and FTSP [8] with an accuracy of 1.5 *μ*s suppress this nondeterminism by time stamping at the media access control (MAC) layer, but they inherently require special MAC implementations.

On the other hand, we have explored into an illumination-based synchronization for vision sensors with a satisfactory accuracy of 28-*μ*s jitters [9]. With our approach, an intensity-modulated light source is employed as an emitter of the synchronization trigger signal, and the imager of the vision sensor itself serves as the receiver of the signal. It requires no communication media other than the visual information, and thus is applicable even to vision sensors equipped with no communication capability but with only data storage for image or image-feature sequences.

**Figure 1.**
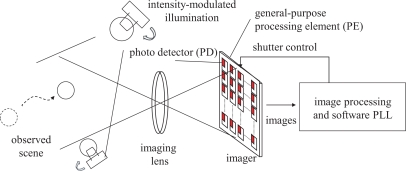
Conceptual diagram of the proposed illumination-based synchronization.

The proposed algorithm is based on the phase-locked loop (PLL) technology [10,11]. Figure 1 illustrates the conceptual diagram of the proposed system. Incident light to the vision sensor serves as the reference signal for synchronization. Internal functions of the vision sensor, including the analog photo integration process in the imager and digital computation executed outside of the imager, forms a PLL to regulate the output signal, which corresponds to the vision frame timing, so that the output is synchronized with the reference.

This phase-locked imaging method essentially relies on the time correlation of the reference and the output signals to regulate the relative phase difference between both signals to a certain value by way of negative feedback, as reviewed in Section 2 in more details. Since the time correlation depends on the relative phase difference and also on the amplitudes of the two signals, we need to know the amplitudes of the signals to ensure that the stability of the control of the phase difference.

However, knowing a priori the amplitude of the reference signal is in general difficult. Because the reference signal emitted by the light source is reflected by the scene and then received by the imager, the spacial configuration of scene objects, the light source and the vision sensors must be known. Moreover, since synchronization of vision sensors are needed when one observes dynamic scenes, this configuration, and thus the amplitude of the reference signal, is dynamically changed due to the motion of scene objects, the light source and the vision sensors. Therefore, we need to estimate the reference amplitude in real time and normalize the signal with respect to this estimated amplitude.

It should be noted that simply taking the time average of image brightness does not give correct estimation of the reference amplitude because there may be background light whose intensity is also unknown. In this paper, we apply the quadrature detection technique to our method in order to separate the reference signal component from the background light. The quadrature detection is a common technique for recovering the amplitude and the phase with lock-in measurement [12]. Our contributions are to integrate it into the frame-based operation of a normal vision sensor and to show that it works even in the cases with shorter photo integration time than the full frame time.

This paper is a revised and extended version of our two conference papers [13,14], one of which is originally proposed to introduce the quadrature technique, while the other introduced an improved per-frame feedback law that enables faster convergence. The remainder of the paper is sequenced as follows: in Section 2 our previous synchronization algorithm is shortly reviewed. In Section 3 the new signal normalization algorithm with the improved feedback law is presented. Section 4 describes the MATLAB simulation and the performance analysis. In Section 5, vivid experimental results with a real high-speed vision sensor in dynamic scenes are presented. Section 6 is the discussion part, where the pros and cons of the signal normalization method is analyzed, and possible solutions and workarounds are proposed. Finally, conclusions are drawn in Section 7.

## 2. Synchronization Algorithm

**Figure 2.**
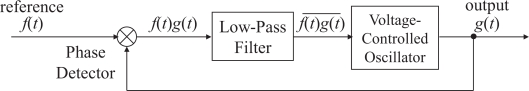
Block diagram of a PLL.

**Figure 3.**
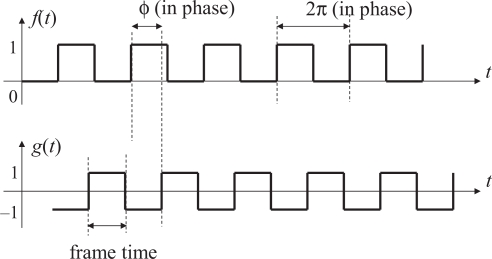
The reference signal *f*(*t*) and the output signal *g*(*t*) in the illumination-based synchronization system.

Figure 2 shows a standard PLL system in which the output signal *g*(*t*) is synchronized to the reference *f*(*t*) in phase as well as in frequency. In the illumination-based synchronization system we proposed [9], *f*(*t*) is the brightness of an intensity-modulated light source with 50% duty ratio and *g*(*t*) is defined as a time function such that *g*(*t*) = 1 when the frame number index of the vision sensor is odd and *g*(*t*) = −1 when even. Reflecting the fact that light intensity cannot take negative values, *f*(*t*) is defined as a square wave taking values 0 and *L*, where *L* is the amplitude of the reference signal, as shown in Figure 3.

Exploiting that *g*(*t*) is a constant during a frame period, the time correlation $\bar{f(t)g(t)}$ can be computed as

(1) $$\bar{f(t)g(t)}\;\propto \;\sum_{i}{\left(-1\right)}^{i-1}F[i]$$

where *i* is the frame number index and *F* (*i*) is the sum of the pixel values obtained within the frame *i*. Because this computational method enables us to compute the time correlation from the images, we do not need any special pixel structures like previous proposals [15,16] in which the produce *f*(*t*)*g*(*t*) is computed within a pixel.

**Figure 4.**
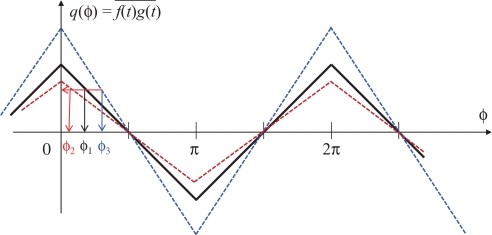
The relation between the time correlation and the phase difference.

The black plot in Figure 4 shows the time correlation *q*(*ϕ*) seen as a function of the relative phase difference *ϕ* between *f*(*t*) and *g*(*t*). For the proposed system, when *q*(*ϕ*) is positive, the frequency of *g*(*t*), and therefore the frame rate, is decreased so that its phase will be lagged. When negative, correspondingly, the frame rate is increased. As long as the feedback characteristic of this loop is properly designed, the system will be converged to the stable equilibrium point *q*(*ϕ*) = *π*/2. This is called the locked state. It also works fine even in the cases with photo integration time shorter than the full frame time [9].

However, it has no adaptability to the changes of the reference signal amplitude *L*. Since the time correlation is proportional to *L*, information about *L* is required to design the feedback system appropriately. This situation is intuitively illustrated in Figure 4. The red and blue dashed lines show the cases with smaller or larger reference amplitudes than the black line case, respectively, but different phase differences *ϕ*_1_, *ϕ*_2_ and *ϕ*_3_ for these three cases yield the same time correlation value.

This phenomenon indicates that the reference amplitude *L* must be known beforehand for appropriate design of the system, but this prerequisite is not always obtainable. This issue becomes more severe when we observe dynamic scenes where *L* will change dynamically. It is showed [9] that moderate fluctuation of *L* can be tolerated, but this imperfection still inevitably limits the practical applicability of the proposed technique.

## 3. Normalization of the Reference Amplitude

### 3.1. Signal Normalization by Quadrature Detection

In order for the synchronization system to work in real environments involving dynamic changes and fluctuations of the received reference amplitude, we propose a method for estimating the amplitude and thereby normalizing the signal. Estimating the reference amplitude is not a trivial task because the imager also receives unknown background light components other than the reference signal.

We introduce the quadrature detection technique to selectively recover the reference amplitude only. Instead of defining a single output signal *g*(*t*), we define an output signal *g*_1_(*t*) and its quadrature counterpart *g*_2_(*t*), which are used to generate two time correlation values $\bar{f(t)g_{1}(t)}$ and $\bar{f(t)g_{2}(t)}$. The quadrature output signal *g*_2_(*t*), however, has to change its value in the midst of a frame and thus the stratagem shown in Equation (1) cannot be used. To avoid this situation, we change the ratio of the reference frequency and the frame rate from 1:2 to 1:4 so that a one-frame time shift on the vision sensor side corresponds to a 90-degree phase shift on the reference side.

Consequently, a straightforward strategy is to define *g*_1_(*t*) so that it takes values 1, 1, −1 and −1 during the frames whose frame number mod 4 are equal to 0, 1, 2 and 3, respectively, and to define *g*_2_ to take −1, 1, 1 and −1 during the corresponding frames. Using the time correlation values $q_{1}\;=\;\bar{f(t)g_{1}(t)}$ and $q_{2}\;=\;\bar{f(t)g_{2}(t)}$, we can estimate that the reference amplitude is proportional to |*q*_1_| + |*q*_2_|, by which the normalization is possible.

Unfortunately, this is not the best choice when we account for the existence of a non-photo-integration period within a frame time. In the locked state with the above definition, a rising or falling edges of the reference signal comes just between a frame and a frame, which is in most cases within a non-integration period. Since no measurement is done in a non-integration period, it is impossible to distinguish the locked state from any situations in its neighbor-specifically the situations where all of the rising and falling edges are within non-integration periods. In other words, the time correlation is not sensitive to the small phase error around *ϕ* = *π*/2. This limits the accuracy of synchronization severely.

**Figure 5.**
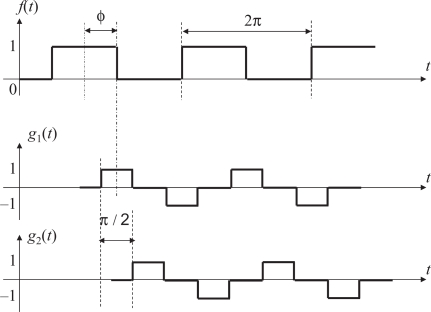
Reference signal *f*(*t*), output signal *g*_1_(*t*), and its quadrature counterpart *g*_2_(*t*).

We address this issue by defining the output signal pair *g*_1_ and *g*_2_ as return-to-zero (RZ) line codes in Figure 5, which means that a separate clock does not need to be sent alongside the signal. One of the output signal *g*_1_(*t*) takes values 1, 0, −1, and 0 during the frames whose frame number mod 4 are equal to 0, 1, 2, and 3, respectively, and the other signal *g*_2_ takes 0, 1, 0, and −1 during the corresponding frames.

**Figure 6.**
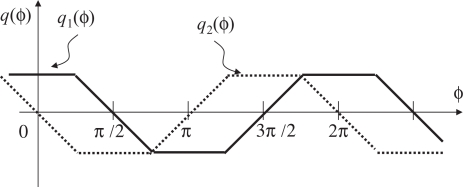
The relations between time correlations and phase difference.

The time correlation values *q*_1_(*ϕ*) and *q*_2_(*ϕ*) seen as functions of the relative phase difference *ϕ* are shown in Figure 6 for the case with full exposure time. Here, the relative phase difference is defined to be zero when the midst time of the reference ‘on’ period and the midst time of the integration period in the frame where *g*_1_(*t*) = 1 coincides. By using the correlation *q*_1_ for feedback, the system can converge to the unique stable equilibrium point *ϕ* = *π*/2. From Figure 6, we can also note that max(*q*_1_(*ϕ*), *q*_2_(*ϕ*)) does not depend on *ϕ* and gives a value proportional to the reference amplitude. By computing this value in real time along with *q*_1_, normalization of the signal amplitude is possible.

**Figure 7.**
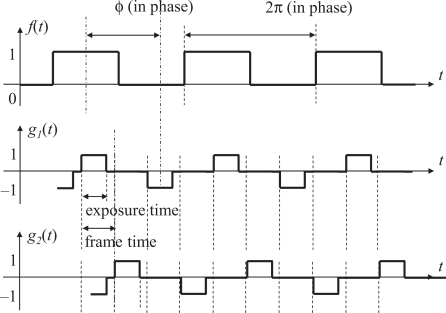
Reference signal *f*(*t*), output signal *g*_1_(*t*), and its quadrature counterpart *g*_2_(*t*), with non-integration time.

**Figure 8.**
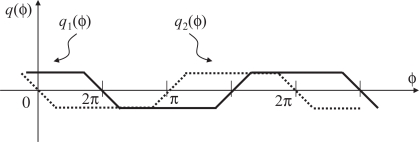
Relation between time correlation and phase difference with non-integration time.

By adopting this definition, the system works well in the cases with shorter integration time. This is essentially because the rising and the falling edges of the reference signals are within integration periods when the system is in the locked state. The relation between the correlation values *q*_1_(*ϕ*), *q*_2_(*ϕ*) and the phase difference *ϕ* when we have a shorter integration period than the full frame time is shown in Figure 7. Clearly, in Figure 8 the correlation *q*_1_ is sensitive to the phase error at the locked state *ϕ* = *π*/2. The quantity for the normalization max(*q*_1_(*ϕ*), *q*_2_(*ϕ*)) still does not depend on *ϕ* and is proportional to the reference amplitude. It should be noted that max(*q*_1_(*ϕ*), *q*_2_(*ϕ*)) also depends on the length of the photo integration period. For reliable normalization, therefore, the length of the photo integration time of the vision sensor must be obtained, or at least it should be the same length as the one used when the feedback gain is designed. This is a reasonable assumption for a vision sensor whose frame time is controllable.

### 3.2. Feedback Algorithm

In our previous implementation [9], the frame time is updated every two frames because one reference period consists of two vision frames. In our new quadrature signal design, however, four vision frames corresponds to a reference period, and a straightforward implementation with per-four-frames update produces a too low control rate. In this section, we describe an algorithm for per-frame feedback suited for our quadrature detection.

Firstly, we apply an equally-weighted moving average filter to the values obtained from consecutive four frames. Consequently, the result is injected into a recursive low-pass filter, of which the coefficients can be designed independently from the four-frame averaging.

For each output frame, after an image is acquired and *F*(*i*) is computed, *F*(*i*) is stored in one of these four variables, *E*_1_, *E*_2_, *H*_1_, and *H*_2_, which are updated according to the following laws:

(2) $$E_{1}\;=\;F[i],\;\text{when }(i\;\text{mod}\;4)\;=\;0$$

(3) $$E_{2}\;=\;F[i],\;\text{when }(i\;\text{mod}\;4)\;=\;1$$

(4) $$H_{1}\;=\;F[i],\;\text{when}\;(i\;\text{mod}\;4)\;=\;2$$

(5) $$H_{2}\;=\;F[i],\;\text{when}\;(i\;\text{mod}\;4)\;=\;3$$

Using these variables, in each frame, the discrete-time low-pass filters to give the time correlations *q*_1_[*i*] and *q*_2_[*i*] at frame *i* are implemented as simple first-order recursive filters with a four-frame moving window

(6) $$q_{1}[i]\;=\;a_{1}\;\cdot \;q_{1}[i-1]\;+\;a_{2}\;\cdot \;(E_{1}\;-\;H_{1})$$

(7) $$q_{2}[i]\;=\;a_{1}\;\cdot \;q_{2}[i-1]\;+\;a_{2}\;\cdot \;(E_{2}\;-\;H_{2})$$

where *q*_1_[*i*] and *q*_2_[*i*] are achieved at the end of every frame. The length of the non-integration period *τ*_nonint_ is negative-feedback controlled every frame in accordance with the time correlation *q*_1_[*i*] and the *normalizer* max(|*q*_1_[*i*]|, |*q*_2_[*i*]|), while the length of the integration period is fixed.

**Figure 9.**
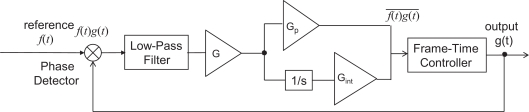
Block diagram of the PLL with PI controller.

We also take into account the frequency mismatch between the oscillator that drives an illumination and the one that drives a vision sensor, by which the central frequency of the vision sensor does not strictly agree with quadruple of the frequency of reference signal. This mismatch causes steady-state residual phase error even in the locked state. In order to remove this error, a PI (proportional-integral) controller is applied to the negative feedback in Figure 9. Specifically, an integral term is added to Equation (8) as

(8) $$\begin{array}{ll} \tau_{\text{nonint}}[i]\;=\; & \tau_{0}\;+\;\frac{G_{p}\;\cdot q_{1}[i]}{\text{max}(|q_{1}[i]|,\;|q_{2}[i]|)}\\ & +\;G_{i}\sum_{j=0}^{\infty }\frac{q_{1}[i-j]}{\text{max}(|q_{1}[i-j]|,\;|q_{2}[i-j]|)}\; \end{array}$$

where *τ*_0_ is a constant set to 0.2 ms, and *G*_p_ and *G*_i_ are constant values explored in the simulation and the experiment section later. The resolution of adjustment of *τ*_nonint_ is 100 ns, which is the instruction cycle of the system used in the experiment chapter.

### 3.3. Effect of Background Light

Until now, we have assumed that there is no background light, which will not be supposed in most realistic situations, as discussed in [9]. By taking into account that our algorithm always takes the difference of the imager output of two successive frames, we can expect that the background light component will be perfectly canceled unless the changes between the background light and the scene reflectance is too large, compared to the modulation frequency. This discussion is validated in the following experiments section.

The advantages of the signal normalization technique also guarantee the deployment of mobile cameras, such as pan and tilt cameras, because when the motion of the mobile cameras is relatively slow compared to the modulation frequency, the amplitude fluctuation of the reference illumination due to angular adjustment of mobile cameras can be ideally eliminated. Similarly, when the whole background is in motion, even if highly dynamic brightness changes occur, the effect cannot strike the successful synchronization result. Undoubtedly, it is not problematic as long as high speed measurement is of interest. Therefore, all kinds of the amplitude fluctuations of the reference illumination can be successfully eliminated owing to the signal normalization algorithm.

## 4. Simulation Results and Performance Evaluation

### 4.1. Sinusoidal Envelope

This chapter presents the simulated results and the evaluated system performance by analyzing the system behavior. The purpose of the simulations is twofold. We aim at exploring feasible parameters for the system while evaluating the performance of synchronization algorithm. We model the visual measurement employing a high-speed vision sensor with 1,000-Hz frame rate and 64 × 64 pixels, which requires 250-Hz modulated illumination according to the central frequency of the normalization algorithm. The frame rate and the number of pixels are decided so that they are equivalent to those of the vision sensor used in the real world experiments in the next chapter.

One of the properties of our algorithm is that no special pixel structure is required, therefore it can be expanded to ordinary vision sensors. More precisely, the only computation depending on the image size is the summation of pixel values, which is not computation consuming. The images can be safely subsampled when the summation is taken, by reading and summing every other pixel to achieve half computational cost. The scalability of the vision system can be accomplished by resetting the pixel coefficients in both simulation and experiment programs with respect to the state-of-art vision sensors, such as 128 × 128 pixels vision chip [17] and the 320 × 240 pixels vision chip [18], and makes the system entirely reconfigurable.

**Figure 10.**
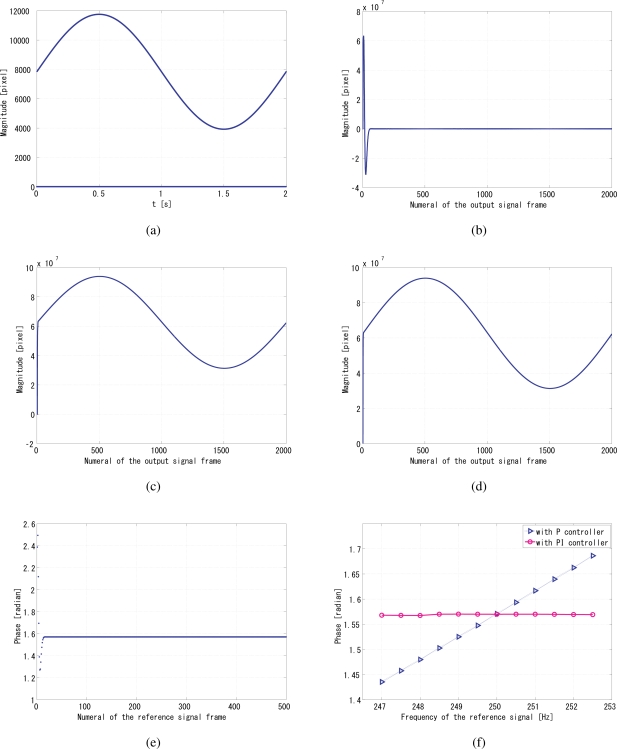
Simulation results with a slow sinusoidal envelope curve, **(a)** *f*(*t*); **(b)** *q*_1_(*t*); **(c)** *q*_2_(*t*); **(d)** maximum; the *normalizer*; **(e)** convergent phase; **(f)** comparison of the steady-state residual phase errors of different reference frequencies.

The coefficients *a*_1_ and *a*_2_ are set to 0.25 and 0.75 respectively, by analyzing the convergence time and the jitters, as well as the undershoot and overshoot of system before convergence, as discussed in [9]. The unit of gain is s/pixel, because in Equation (8) *q*[*i*] is in the dimension of the pixel value multiplied by the number of pixels, and the pixels value is dimensionless. Figure 10 (a) shows the reference signal with a slow sinusoidal envelope curve, when the gain *G*_p_ was set to 550, and *G*_i_ was set to 32. Figure 10 (b) shows the time correlation value of the output signal *g*_1_(*t*), and Figure 10 (c) shows the time correlation value of its quadrature counterpart *g*_2_(*t*), while Figure 10 (d) shows the maximum between *q*_1_ and *q*_2_, which is the *normalizer*. Divided by this *normalizer*, the correlation value *q*_1_ is normalized, equivalently the gain can be maintained as a constant value. Figure 10 (e) shows the relative phase of the output signal to the reference signal. It can be seen that the system immediately converged to the *π*/2 relative phase and became stable thereafter. Figure 10 (f) illustrates the comparison of the steady-state residential phase error of different frequencies of the reference signal. Apparently, the PI feedback helps to reduce the discrepancy between *π*/2 and the real phase, which corresponds to the steady-state error. Conceptually, the jitters were evaluated for the duration between the convergence time and 2 s from the beginning of the measurement. Leastwise, the relative phase between the reference and output is very stable and on the order of 10^−3^ rad within this range of gains.

**Figure 11.**
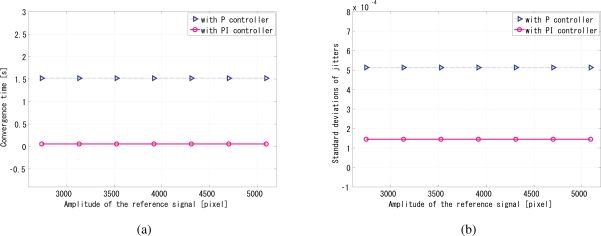
Synchronization is independent of the amplitudes of reference signal. **(a)** convergence time of different amplitudes of the reference signal; **(b)** jitters of different amplitudes of the reference signal.

Previously, the original algorithm without signal normalization technique cannot tolerate too large amplitude variations of reference signal. Actually, when 5-Hz sinusoidal signals with different amplitudes added to a constant direct-current (DC) synchronization was successful only as long as the sinusoidal amplitude is within 7.75% of the DC component. The synchronization performance is analyzed in Figure 11, in which the convergence time and jitters are completely not dependent of the amplitudes of reference signal in a feasibly wide range. Furthermore, the per-frame negative feedback algorithm greatly reduces the convergence time on the order of 1 s in Figure 11 (a), while the jitters stay almost the same on the order of 10^−4^ rad in Figure 11 (b).

**Figure 12.**
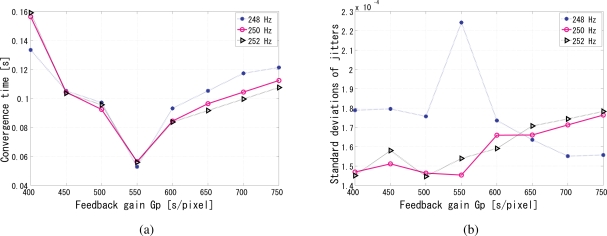
Jitter analysis of the simulation results, **(a)** convergence time of different *G*_p_; **(b)** phase jitters of different *G*_p_.

**Figure 13.**
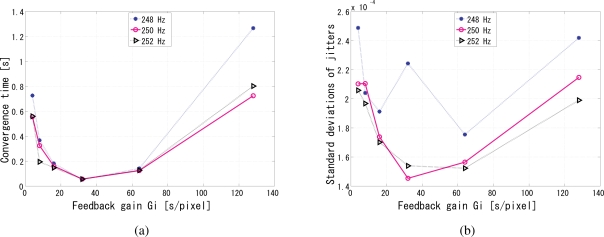
Jitter analysis of the simulation results, **(a)** convergence time of different *G*_i_; **(b)** phase jitters of different *G*_i_.

The synchronization performance is analyzed in Figure 12 for different gains of the PI controller, *G*_p_ and *G*_i_ respectively. In these simulations, we also tested reference signal frequencies slightly different from 250 Hz, to simulate the possible mismatch between the nominal and the actual operating frequencies in every clock oscillator, and the simulation results go on well. Figure 12 (a) shows the convergence time for different gains and reference frequencies. Apparently, for the central frequency, it indicates that the *G*_p_ around 550 results in stable convergence time for this setup, while comparably the smallest jitters in Figure 12 (b). The trends are almost the same for the frequencies slightly drifting from it. The discrepancies in the reference frequency did not cause significant changes in the convergence time. The ratio of the upper and lower limits of the gain with which the system converges was approximately twenty, which means the system works well for a reasonably wide range of gains. Figure 12 (b) shows the jitters for different gains and reference frequencies. Leastwise, the relative phase between the reference and output is very stable and on the order of 10^−3^ rad within the feasible range of gains. The analysis is the same for *G*_i_. When *G*_i_ is 32, both the convergence time in Figure 13 (a) and the jitters in Figure 13 (b) reach the minimum.

### 4.2. Real-World Scene

Secondly, we use the signal normalization algorithm to guarantee the assumption that even if the average brightness of a scene changes rapidly and randomly, is not restrictive for the proposed method to be applied to realistic visual measurement. In order to attest this, we use a brightness sequence from a real scene as the envelope of the reference input, where there is no self-luminous objects, but only the strongly reflected light.

**Figure 14.**
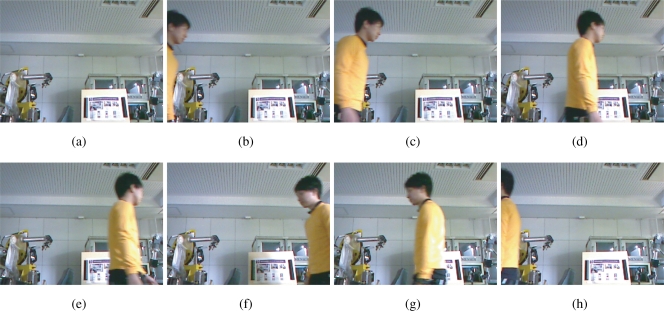
The real world scene whose brightness sequence is used as the envelope of the input for simulation, which assumes a surveillance scenario, **(a)** 0 s; **(b)** 1.523 s; **(c)** 1.749 s; **(d)** 2.259 s; **(e)** 2.578 s; **(f)** 4.916 s; **(g)** 5.415 s; **(h)** 6.179 s.

**Figure 15.**
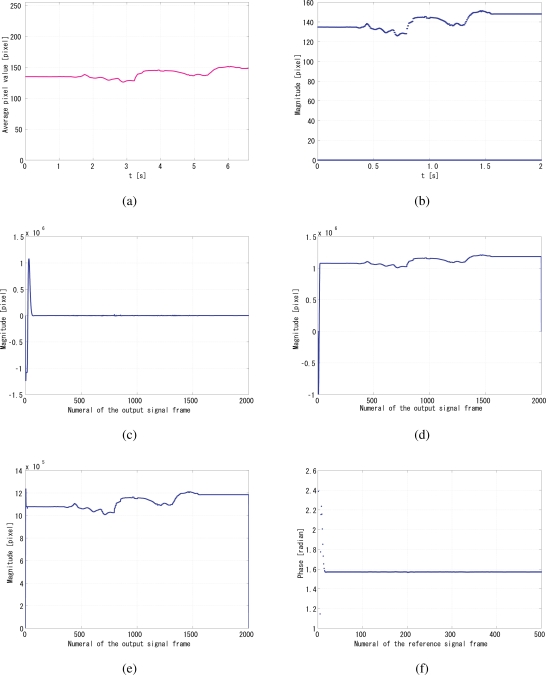
Real-world simulation results, **(a)** average pixel values per frame; **(b)** *f*(*t*); **(c)** *q*_1_(*t*); **(d)** *q*_2_(*t*); **(e)** maximum; the *normalizer*; **(f)** convergent phase.

Figure 14 shows the snapshots of an indoor scene in which a person is walking around within the field of view of a camera (Logicool Qcam S 7500, 640 × 480 pixels, 30 Hz, 8 bits). The average of the pixel values within each frame is computed by the computer vision library OpenCV to obtain the average brightness sequence of the scene *b*(*t*), which is shown in Figure 15 (a). Note that the scene includes a large reflective moving region so that the average pixel values fluctuate randomly and abruptly. The reference signal *f*(*t*) for the simulations is generated from *b*(*t*) and the 250-Hz unity-amplitude square wave *r*(*t*) as *f*(*t*) = *Nb*(*t*)*r*(*t*), where *N* = 64 × 64 is the number of the pixels. This corresponds to the situation that all of the room light of this scene is replaced with the intensity-modulated illumination and no self-emissive light source exists.

Figure 15 shows a successful case. Figure 15 (b) shows the reference signal injected with the real-world average pixel values, when the gain *G*_p_ was set to 550, and *G*_i_ to 32. Figure 15 (c) shows the time correlation value of the output signal *g*_1_(*t*), and Figure 15 (d) shows the time correlation value of its quadrature counterpart *g*_2_(*t*), while Figure 15 (e) shows the maximum between *q*_1_ and *q*_2_. This maximum is the *normalizer*. Figure 15 (f) shows the relative phase of the output signal to the reference signal. It can be seen that the system immediately converged to the *π*/2 relative phase and became stable.

## 5. Experiments

### 5.1. Experimental Setup

**Figure 16.**
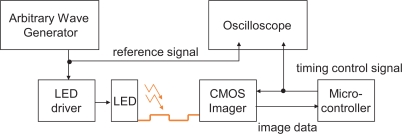
Block diagram of the experiment setup.

To demonstrate the practical utility of the proposed method, synchronization was implemented on a real vision sensor. We employed a high-speed vision system called VCS-IV developed by the authors [19], which captures and processes images in real time at the frame rate up to around 1,000 Hz. The VCS-IV vision system is equipped with a 64 × 64-pixels CMOS imager called *Digital Vision Chip*, which has capability of pixel-parallel image processing programs on the focal-plane processing element array. This capability is not utilized in the presented experiment except for computation of summation of 6-bits digital pixel values over the array, which is used as *F* [*i*] in the same way as done in simulation. Figure 16 shows the block diagram of the experimental setup.

The illumination system consists of a Nissin Electronics LDR-90 LED array and an LPR-30W-D power supply system, which are driven by the reference square-wave signal from a Tektronics AFG3102 arbitrary wave generator. LED light can be easily modulated at a high frequency and therefore is selected as the illumination source [20,21]. It is modulated by the wave generator to be a continuous series of 250 Hz square wave, and can be displayed on the oscilloscope as the reference signal.

The operation of this high speed vision system was measured by observing the pixel reset signal of the imager, whose positive edge corresponds to the beginning of an integration period, by a Tektronics TDS3034 oscilloscope. If the operation of the vision system is locked to the illumination, synchronized waveforms of the pixel reset and the reference signal will be observed in the oscilloscope. The blue signal is the reference signal, while the yellow one locked to it is the output signal.

### 5.2. Experimental Results

**Figure 17.**
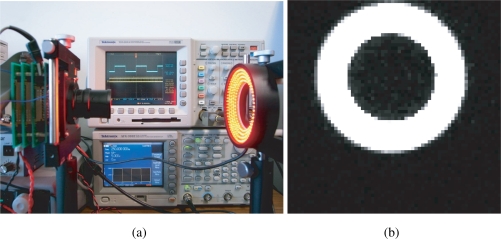
**(a)** spontaneous operations of the vision chip; **(b)** a real-world scene image taken by the vision chip.

**Figure 18.**
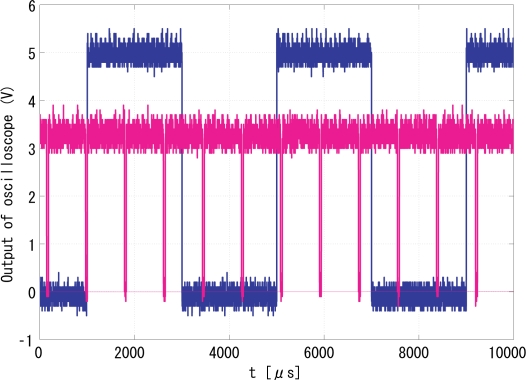
Output signal compared to the reference signal without synchronization.

**Figure 19.**
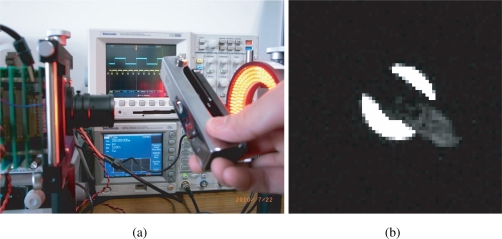
**(a)** direct illumination; **(b)** image of a reflective moving gadget.

**Figure 20.**
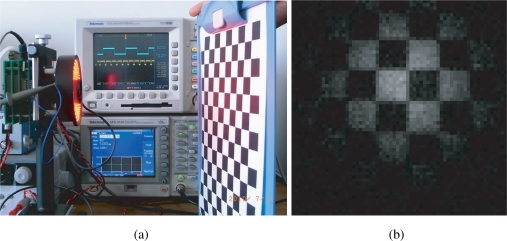
**(a)** indirect illumination; **(b)** image of a reflected checkerboard.

The experiment results are generalized in Table 1. When the synchronization is not successful due to inappropriate gains, although the vision chip can take real world images as usual in Figure 17, the spontaneously electronic shutters of the vision chip are not locked to the reference signal in Figure 18. More precisely, feedback control is not executed during the non-integration time. Items 2–6 describe the various successfully synchronized cases. The LED directly shed light on the vision system with a reflected gadget in a normal laboratory environment is shown in Figure 19 (a). The movement and the reflected light of the gadget before the lens do not disturb the synchronization at all. The illuminance measured in front of the imaging optics when the LED light is off was 187 lx, and was 3460 lx when the LED is on without intensity modulation. An image of the gadget taken by the vision system is shown in Figure 19 (b). When the reference signal is an indirect illumination, such as reflected light in Figure 20 (a), about 1068 lx, with free changes of direction the synchronization is also successful owing to the signal normalization algorithm. An image of the checkerboard taken by the synchronized vision chip is shown in Figure 20 (b). Actually, reflected light is the most common source of illumination. The upper limit of synchronization distance is 1 meter under 3460 lx. The LED brightness can be freely adjusted within the range from 142 lx to 3850 lx, corresponding to the changes of orientation of light source. Note that the imager used here has considerably low sensitivity and is noisy, and the background texture is almost unobservable. The average pixel value over both of the illuminated and unilluminating pixels during a 50-frames sequence was 18.4 with 0.053 standard deviation, while it was 0.8 with 0.036 standard deviation when the LED is off.

**Figure 21.**
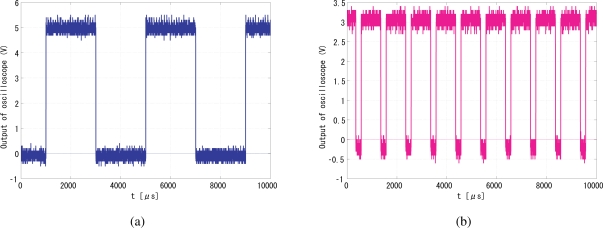
Successful experimental results, **(a)** input signal; **(a)** output signal.

**Figure 22.**
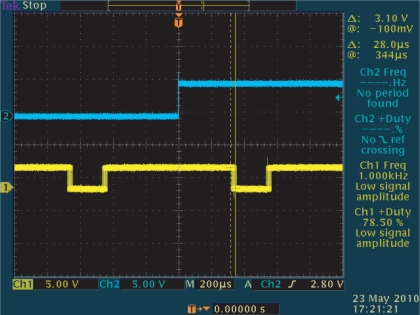
Peak-to-peak jitters of the output signal, 28-*μ*s.

Figure 21 (a) shows snapshots of the 250-Hz square-wave signal to drive the LED and the output (pixel reset) signal Figure 21 (b) that successfully locked to the illumination reference, where *G*_p_ and *G*_i_ are set to 256 and 64, respectively. The coefficients of the low-pass filter in Equations (6) and (7) are also empirically optimized to 0.5 and 0.125. The output signal was synchronized to the reference signal with *π*/2 relative phase shift and twice the frequency. The peak-to-peak jitters of the output signal measured by the oscilloscope as shown in Figure 22, was around 28-*μ*s in either normal condition or dynamic scenes, which is only 1.0% of the reference period and thus 0.1-rad phase error. This 0.1-rad phase error is satisfactory enough for practical utility.

## 6. Discussion

A high performance illumination-based synchronization algorithm normalizing the amplitude of the reference signal employing per frame negative feedback is proposed and evaluated. Both simulated and experimental results attest the advantage of new algorithm. Amplitude fluctuations caused by the reflected illumination, the passengers shadows, or the brightness adjustment of illumination source can be tolerated by normalizing the amplitude of the reference signal every frame by the maximum value of the time correlation between the reference signal and its quadrature counterpart. As demonstrated in the MATLAB simulation, with the help of PI controller, frequency mismatches between the central frequency, caused by either congenital machine error or human operation error, can be successfully eliminated. In the real world experiment, the advanced algorithm successfully removes the disturbance of all sorts of amplitude fluctuation of the LED illumination, greatly reduce the peak-to-peak jitters of the output signal, and the steady-state residual phase error has been consequently eliminated.

However, there are some intrinsic weaknesses in this method along with the all strengths discussed above. Firstly, the visibility of scene has been affected by the new intensity modulation method. In the previous algorithm, the visibility was never interfered because the half period of every frame is always illuminated in the locked state. Comparably, in the signal normalization algorithm, due to the intensity modulation method with the help of quadrature detection, in the locked state, there is one vision frame among four frames that will not completely be illuminated. This phenomenon certainly has inconvenient influence in practicability. To ensure the visibility, background light is requisite. Furthermore, compensation for the brightness between frames is required when analyzing the video sequence taken by this method, which will be investigated in the future work.

Meanwhile, the relationship between the reference frequency and the frame rate has been changed. In practical point of view, this signal normalization method is a two-edged sword. Because the quadruple frequency relation between the frame rate and the reference signal may give rise to the blinking annoyance to human beings, such as a 25 Hz blinking illumination is a necessity to synchronize vision sensors operating at 100 Hz. However, this disadvantage can be solved by employing invisible illumination, such as infrared light.

## 7. Conclusions

An advanced illumination-based synchronization algorithm employing signal normalization technique based on PLL for high-speed vision sensors has been described. The influence of the amplitude fluctuation of the illumination signal can be effectively eliminated with no steady phase error and minimum peak to peak jitters. Both simulated and experimental results demonstrate that the electronic shutters of sensor can be successfully locked to the LED illumination signal even under significant disturbance of the reference brightness in real-world environment. Both direct and indirect illumination can be employed as the reference signal and therefore guarantee the practical application of this visible light communication technique.

## Figures and Tables

**Table 1.** Experiment conditions and results

	**Optical Source**	**LED Illuminance**	**Effective Distance**	**Jitters**	**State**
1	direct illumination	3460 lx	N/A	N/A	unlocked
2	direct illumination	3460 lx	1.0 m	24	locked
3	moving reflective gadget	340 lx	1.0 m	28	locked
4	indirect illumination	1068 lx	0.8 m	28	locked
5	direct illumination	142 lx	1.0 m	28	locked
6	direct illumination	3850 lx	1.5 m	28	locked
